# The distribution of interstitial cells of Cajal in congenital ureteropelvic junction obstruction

**DOI:** 10.1007/s11255-013-0454-7

**Published:** 2013-04-30

**Authors:** Wojciech Apoznanski, Piotr Koleda, Zdzislaw Wozniak, Leslaw Rusiecki, Tomasz Szydelko, Dariusz Kalka, Witold Pilecki

**Affiliations:** 1 Department and Clinic of Paediatric Surgery and Urology, Wroclaw Medical University, ul. M.Sklodowskiej-Curie 50/52, 50-369 Wrocław, Poland; 2 Department of Pathophysiology, Wroclaw Medical University, ul. K.Marcinkowskiego 1, 50-368 Wrocław, Poland; 3 Department of Pathomorphology, Wroclaw Medical University, ul. K.Marcinkowskiego 1, 50-368 Wrocław, Poland; 4 Department of Palliative Care Nursing, Wroclaw Medical University, ul. K.Bartla 5, 51-618 Wrocław, Poland

**Keywords:** C-kit, Distribution, ICC, Interstitial cells of Cajal, Obstruction, Ureteropelvic junction

## Abstract

**Purpose:**

The authors analysed the distribution of c-kit-positive interstitial cells of Cajal (ICCs) in obstructed ureteropelvic junction (UPJ) and its age-related changes.

**Methods:**

Twenty specimens were obtained from children with intrinsic ureteropelvic junction obstruction (UPJO), at the average age of 8.1 years (8 months–16.8 years), fixed in formalin and embedded in paraffin. Five control samples were taken from children at the average age of 2.3 years (2.4 months–7.4 years). All specimens were analysed by the immunohistochemistry test with light microscopy with respect to c-kit expression. The distribution of c-kit-positive ICCs in the two groups was compared and the correlation between the distribution of c-kit-positive ICCs and the patients’ age in UPJO cases was analysed. The results were examined by Yates’ χ^2^ test, Mann–Whitney *U* test, and *t* test for Pearson’s correlation coefficient. A *P* value < 0.05 was considered as statistically significant.

**Results:**

No statistically significant differences were found in the distribution of c-kit-positive ICCs between UPJO and the control group. No correlation was established between the age of patients with UPJO and the distribution of c-kit-positive ICCs.

**Conclusion:**

No distributional difference found in obstructed and unobstructed UPJ seems to indicate that UPJO is not associated with anomalous distribution of c-kit-positive ICCs. Age-related changes in the expression of c-kit-positive ICCs are equally distributed in obstructed UPJ.

## Introduction

Congenital ureteropelvic juncture obstruction is the most common pathology of the upper urinary tract resulting in hydronephrosis in children. The aetiology of ureteropelvic junction obstruction (UPJO) remains unknown. Recent research suggests that the cause of the obstruction may be functional disorders and not, as previously assumed, anatomic defects [1–3]. Recent investigations have concentrated on interstitial cells of Cajal (ICCs), whose presence was detected in the urinary tract. Numerous researchers emphasise the role of interstitial cells of Cajal in modulating the transmission of peristaltic waves across ureteropelvic junction (UPJ) [4–12]. The expression of the c-kit receptor tyrosine kinase on the surface of the cells and the positive reaction of ICCs with antibodies to the proto-oncogene c-kit made it possible to identify ICCs in the human urinary tract [4].

A number of reports published in the last decade showed the expression of c-kit- positive ICCs in UPJ in patients with UPJO. Yet the results of the analyses are disparate [5, 10, 12]. The report on c-kit-positive ICCs expression changes in UPJ induced by the ligation of ureters in rats created even bigger stir [13].

Interstitial cells of Cajal in the urinary tract are identified indirectly. Therefore, the analyses of changes in ICC population do not indicate the number of the cells, but merely their presence (c-kit expression). Parallel analyses of ICCs in the alimentary tract in animals demonstrate enormous plasticity of ICCs and may suggest a similar potential for ICCs in the urinary tract [14–16].

Disparate results in the analyses of ICC density as well as the limitations of indirect identification of ICCs made the authors decide to investigate the distribution of active ICCs in UPJ in patients with congenital UPJO. In the authors’ opinion prior to the research, distribution might have been yet another factor, in addition to density, potentially affecting UPJ—predetermining or resulting in UPJO.

## Purpose

The objective of the following study is to thoroughly analyse the distribution of ICCs in UPJ in children with UPJO.

## Methods

### Material

Renal scintigraphy and ultrasound were performed to diagnose the ureteropelvic obstruction. There were 20 cases in the UPJO group (mean age 8.1 years, range 8 months–16.8 years) and 5 cases in the control group (mean age 2.3 years, range 2.4 months–7.4 years) (Table 1). Only cases of intrinsic obstruction confirmed intraoperatively were included in the study. All patients in the control group underwent nephrectomy because of Wilms tumour. They did not present any clinical or radiographic UPJO symptoms and their UPJ were free of tumour cell invasion. In each case, a 5–6 mm sample of ureteropelvic junction was taken for the analysis. The sample was fixed immediately in 4 % formalin with phosphate buffer and embedded in paraffin.

**Table 1:** The comparison of both wide and homogenous UPJO patients with controls

	UPJO group	UPJO homogenous group	Control group
Age (mo.)	8.03–201.37	8.03–62.13	2.43–88.80
Median (mo.)	95.92	14.20	14.53
Arithmetic mean (mo.)	96.95	26.53	27.40
SD (mo.)	63.81	20.33	34.81
Patients no.	20	7	5
Girls	7	5	3
Boys	13	2	2
Girls:boys	1:1.86	2.5:1	1.5:1

*mo.* months, *no.* number, *SD* standard deviation, *UPJO* ureteropelvic junction obstruction

### Immunohistochemistry and light microscopy

**Fig. 1:**
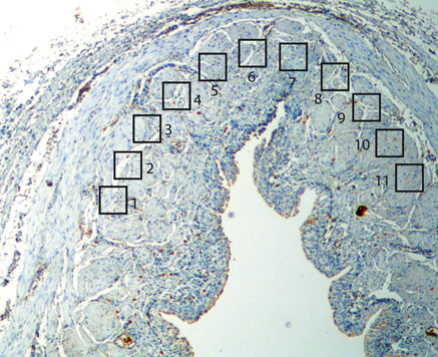
Ureteropelvic junction cross-sectional specimen with 11 neighbour well-stained, oriented high-power fields used for number of ICC gradient assessment. Reduced from ×100

**Fig. 2:**
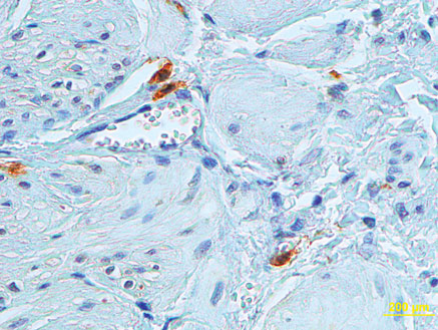
Ureteropelvic junction cross-sectional specimens with c-kit-positive ICCs in the inner border of the circular muscle layer. Reduced from ×400

The details of the applied procedures are to be found in our previous report [5]. In each specimen, 11 neighbour well-stained, oriented high-power fields of 0.136 mm^2^ each were evaluated, and the number of c-kit positive ICCs was counted (Fig. 1). To evaluate the distribution of ICCs in UPJ, the concept of gradient has been introduced, which is defined as a difference bigger than one cell in the number of ICCs in successive adjacent fields of vision. Once the number of ICC gradients in adjacent fields was counted, it was analysed in relation to the patient’s age. The assessment was made by two independent investigators. ICCs were identified in the inner border of the circular muscle layer and were oriented parallel to muscle fibres. ICCs had a fusiform cell body with a thin cytoplasm with a large oval nucleus (Fig. 2).

### Statistics

Results were examined by Yates’ χ^2^ test, Mann–Whitney *U* test, and *t* test for Pearson’s correlation coefficient. A *P* value < 0.05 was considered statistically significant.

### Homogenisation

As the compared groups were not homogenous, we reduced the number of patients in the examined group to 7 cases of UPJO, at the average age of 2.2 years (8 months–5.2 years). The control group remained the same and was constituted by 5 patients at the average age of 2.3 years (2.4 months–7.4 years) (Table 1). Homogenisation made it possible to minimise the age difference to a statistically non-significant value. The results obtained from the homogenous groups were verified statistically with Yates’ chi-square test, Mann–Whitney *U* test, and *t* test for Pearson’s correlation coefficient. A *P* value < 0.05 was considered statistically significant.

## Results

No statistically significant difference was found between the number of gradients (patterns of distribution) of c-kit-positive ICCs in UPJ in patients with UPJO prior to or after homogenisation and in patients from the control group (*P* = 0.3753, *P*_h_ = 0.1689; Tables 2, 3).

**Table 2:** Results of 11 neighbour high-power field assessment for the number of ICC gradients—the comparison of the obstructed UPJ and normal specimens

	Patients no.	ICC gradients no.
UPJO group	20	50
Control group	5	10
Difference (P)		NS

*no.* number, *ICC* interstitial cells of Cajal, *NS* not statistically significant, *UPJO* ureteropelvic junction obstruction

**Table 3:** Results of 11 neighbour high-power field assessment for the number of ICC gradients—the comparison of the UPJO homogenous group and controls

	Patients no.	ICC gradients no.
UPJO homogenous group	7	19
Control group	5	10
Difference (P_h_)		NS

*no.* number, *ICC* interstitial cells of Cajal, *NS* not statistically significant, *UPJO* ureteropelvic junction obstruction

No statistically proven correlation was established between the number of gradients of c-kit-positive ICCs (patterns of c-kit-positive ICC distribution) in UPJ and the age of the patient with UPJO (*P* = 0.087, *R* = −0.3927) (Fig. 2).

**Fig. 3:**
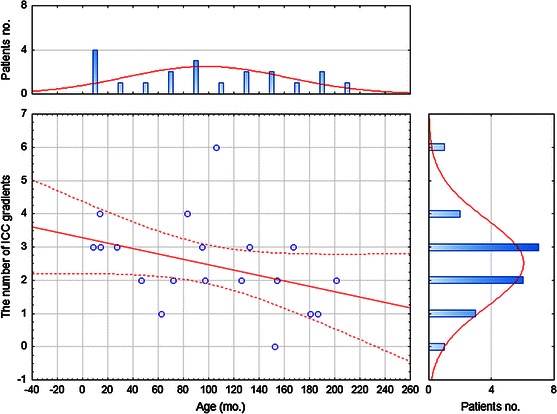
The correlation between age of the patients with congenital ureteropelvic junction obstruction and the number of ICC gradients

It may be concluded that as no differences in the distribution of c-kit-positive ICCs in UPJ between the patients with UPJO and the control group have been found, UPJO is not associated with distributional changes of c-kit-positive ICCs in obstructed UPJ. Age-related changes in the expression of c-kit-positive ICCs do not entail the distributional changes of c-kit positive ICCs in obstructed UPJ, either (Fig. 3).

If alternations in the expression of c-kit- positive ICCs in UPJO are due to the development of a compensatory mechanism for the failure of urine to pass through the upper urinary tract (increased expression) and the regression of compensatory changes once the compensatory mechanism is no longer efficient, then the two processes proceed steadily in UPJO and do not disturb the distributional patterns of c-kit-positive ICCs.

## Discussion

Numerous studies concerned with the expression of c-kit-positive ICCs in the upper urinary tract have been published for the last decade, but no conclusion has been reached. Some researchers maintain that the changes in the expression of c-kit-positive ICCs are the primary causes of UPJO, which is suggested by the decreased density of ICCs observed in a number of studies [10, 12].

Our own results obtained earlier point to a different interpretation. Fields with numerous c-kit-positive ICCs occurred statistically significantly more often in UPJ of patients with UPJO than in the control group. Similarly, fields with few cells were statistically significantly rarer in patients with UPJO than in the control group. The statistical analysis established the correlation between the total number of c-kit-positive ICCs expressed in obstructed UPJ and the patient’s age and indicated that the older the patient the lower the number of ICCs [5].

Bearing in mind that our results are in disparity with the results obtained by other researchers described above, it seems advisable to refer to observations on animal data which may also contribute to the discussion on the nature of ICC expression changes. Ekinci et al. [17] demonstrated increased amplitude and frequency of spontaneous renal pelvic contractility as a consequence of UPJO in rabbits. According to the study, the above increase may be reactive to overactivity and growth of pace-making cells. Kuzgunbay et al. [13] showed that the expression of c-kit- positive ICCs in the acquired ureteral obstruction in a rat was time-related. Immediately following the acquisition of obstruction, the number of c-kit-positive ICCs was increasing to achieve the peak level after 14 days, and then it gradually decreased to reach plateau after 60 days at a statistically significantly higher level than in the control group (with no UPJO). Placebo administration did not affect the expression of c-kit-positive ICCs.

In our opinion, there might be few causes of the disparity among the reports on the expression changes of c-kit-positive ICCs in UPJO. One derives from indirect identification of ICCs on the basis of the expression of the c-kit receptor tyrosine kinase on the surface of the cells. The other problem, which is the consequence of the identification method, might be that ICC plasticity in the urinary tract is still unexplored in contrast to ICC plasticity in the gastrointestinal tract [14–16].

One more issue needs to be discussed in reference to the UPJO group. We built our study group with the patients with an extrinsic (e.g. external compression, crossing vessel, etc.,) UPJ obstruction excluded. We did not evaluate these intrinsic UPJO patients for any functional or other causes of obstruction that might affect ICC expression.

Our study group consists of UPJO patients of different age and different time of pathology duration as well. The time of pyeloplasty and obtaining a UPJ sample was determined by the decreased renal function on renal scintigraphy. We cannot exclude the influence of UPJO duration on ICC expression. Possibly a chronic course of disease with exacerbations or even acute UPJ obstructions my induce ICC expression changes.

Although possible functional UPJ obstruction and different pathology duration may influence ICC expression, both factors being our methods’ limitations, in this study, we did not analysed straight ICC expression, but the pattern of their distribution in UPJO. We focused on abnormalities of ICC distribution and gradients in ICC expression. Gradients show differences in the number of ICC in a specimen between neighbour fields. To minimise age differences between study and control groups, we homogenised them.

In absence of pertinent reports, our main concern was whether ICCs were equally distributed in UPJ and whether UPJO affected their distribution. This study is an attempt to examine the distribution of c-kit-positive ICCs in UPJ. So far, the expression of c-kit-positive ICC has been described in terms of density, that is, the number of c-kit-positive ICCs per high-power field. Such analyses, however, do not reveal distributional patterns. In our opinion, the comparative analysis of specimens from UPJ of patients with and without UPJO which consists in the evaluation of successive adjacent 11 fields in terms gradients, that is, considerable differences in the number of c-kit-positive ICCs, shows the distributional patterns.

No differences in the distribution of c-kit-positive ICCs in UPJ between patients with UPJO, and those from the control group signify that UPJO is not associated with distributional changes of c-kit-positive ICCs in obstructed UPJ. The age-related ICC expression changes previously reported are not connected with distributional changes, either. The findings indirectly suggest that the changes affecting ICCs in obstructed UPJ are regular and steady. Further investigation and other diagnostic methods (e.g. electron microscopic evaluation) may cast more light on the nature of ICCs in the urinary tract.
